# Efficacy and prognosis analysis of pulley traction-assisted endoscopic submucosal dissection with dental floss for early gastric cancer and precancerous lesions

**DOI:** 10.3389/fsurg.2025.1477658

**Published:** 2025-07-21

**Authors:** Hao Luo, Jie Tang, Xiaotong He, Yinglei Shi, Yunli Chang

**Affiliations:** ^1^ Department of Gastroenterology, Shanghai General Hospital, Shanghai, China; ^2^ Department of Gastroenterology, Jiangwan Hospital of Hongkou District, Shanghai, China; ^3^ Department of Gastroenterology, Shanghai Pudong New Area People’s Hospital, Shanghai, China

**Keywords:** gastric cancer, dental floss traction, early stage, precancerous lesions, endoscopic submucosal dissection

## Abstract

**Objective:**

To investigate the clinical efficacy of pulley traction-assisted endoscopic submucosal dissection (ESD) with dental floss in patients with early gastric cancer and precancerous lesions, and its impact on patient prognosis.

**Methods:**

Clinical data of 77 patients with early gastric cancer and precancerous lesions were retrospectively analyzed. The patients were divided into groups according to different treatment regimens; 38 patients in the control group underwent ESD, whereas 39 patients in the study group underwent pulley traction-assisted ESD with dental floss. Lesion resection status, perioperative indicators, gastrointestinal function indicators, complications, and recurrence were compared between the two groups.

**Results:**

The curative and *en bloc* resection rates in the study group were significantly higher than those in the control group, whereas the residual rate of basal lesions was significantly lower than that in the control group (*P* < 0.05). The study group experienced less intraoperative bleeding and had a significantly shorter operative time, anal exhaust time, first eating time, and length of hospital stay (*P* < 0.05). Three days postoperatively, motilin (MTL), gastrin (GAS), pepsinogen I (PG I), and pepsinogen II (PG II) levels were significantly reduced in both groups, with significantly higher levels in the study group (*P* < 0.05). The incidence of complications in the study group was 7.69% (3/39), which was significantly lower than that in the control group [26.32% (10/38); *P* < 0.05]. Three months postoperatively, the quality of life scores (FACT-G) in all aspects significantly increased in both groups, with the study group showing significantly higher scores (*P* < 0.05). The recurrence rate at 12 months postoperatively in the study group (5.13%) was significantly lower than that in the control group (23.68%; *P* < 0.05).

**Conclusion:**

Pulley traction-assisted ESD with dental floss is effective in treating early gastric cancer and precancerous lesions.

## Introduction

Gastric cancer is a malignant tumor with high morbidity and mortality rates, with over one million new cases of gastric cancer and more than 768,000 deaths in 2020 worldwide. The incidence of gastric cancer is particularly high in East Asia, affecting 45.7 individuals per 100,000 people. Although the detection rate of early gastric cancer varies globally, Japan and South Korea report rates exceeding 50%, whereas Western countries, such as the United States, report rates of approximately 20% (1). China, as a nation with a high incidence of gastric cancer, accounts for 44.21% of global cases; however, the detection rate of early gastric cancer is approximately 10%–20%. Early gastric cancer typically lacks apparent symptoms and is often diagnosed at an advanced stage when the clinical manifestations arise, causing patients to miss the optimal window of treatment.

Early gastric cancer refers to a malignant lesion occurring in the mucosal or submucosal layer of the stomach without any evidence of lymph node metastasis. Patients with early gastric cancer are typically asymptomatic and are often diagnosed during routine check-ups (2). Compared to advanced gastric cancer, early gastric cancer exhibits significantly better treatment outcomes, with a 5-year survival rate exceeding 90% following early surgical resection (3, 4). The 5-year survival rate of patients with advanced gastric cancer is typically less than 15%. Even with the combined application of neoadjuvant chemotherapy, molecular-targeted therapy, and immunotherapy, therapeutic outcomes remain limited. As early gastric cancer is typically confined to the gastric mucosa or submucosa, its treatment is relatively simple and can significantly enhance patient survival rates, reduce therapeutic complexity and side effects, and effectively alleviate both the financial burden and psychological stress on patients. Therefore, the early detection and treatment of gastric cancer are pivotal for enhancing survival rates and improving the quality of life of patients.

In recent years, with the continuous advancement of endoscopic techniques, treatment strategies for early gastric cancer and precancerous lesions have become increasingly refined. Endoscopic submucosal dissection (ESD), due to its minimally invasive nature, rapid recovery, and preservation of gastric function, is the preferred approach for treating early gastric cancer and precancerous lesions (5, 6). Meanwhile, a variety of auxiliary techniques have emerged, such as traction-assisted ESD, endoscopic suturing systems, and robotic-assisted operations, which have effectively improved procedural efficiency and safety, while reducing the risk of complications such as perforation and bleeding (7, 8). In addition, the standardization of wound management, postoperative monitoring, and follow-up strategies has further contributed to improving long-term outcomes in patients. However, during ESD, the need for a large field of view makes the surgery susceptible to bleeding, which restricts visibility, increases the complexity of the procedure, and may lead to complications, such as perforation (9, 10). Therefore, further improvement in the therapeutic efficacy of ESD and the reduction of complications have become focal points in contemporary medical research.

In recent years, with the continuous advancement of medical technology, traction-assisted techniques have been increasingly used for ESD, demonstrating promising prospects for application (11). Dental floss has become a focal point in adjunctive therapy research owing to its exceptional elasticity, resilience, ease of use, and low cost (12). Traction-assisted ESD using dental floss can effectively expose the lesion mucosa, expand the surgical field, and aid in the removal of lesion specimens, thereby improving resection outcomes (13). Although this method has been applied to certain gastrointestinal diseases, few reports are available on the use of pulley traction-assisted ESD with dental floss for early gastric cancer and precancerous lesions. Therefore, this study aimed to investigate the clinical efficacy and prognosis of pulley traction-assisted ESD with dental floss for the treatment of early gastric cancer and precancerous lesions, providing a reference for the broader application of this therapeutic approach.

## Materials and methods

### Clinical data

**Figure 1:**
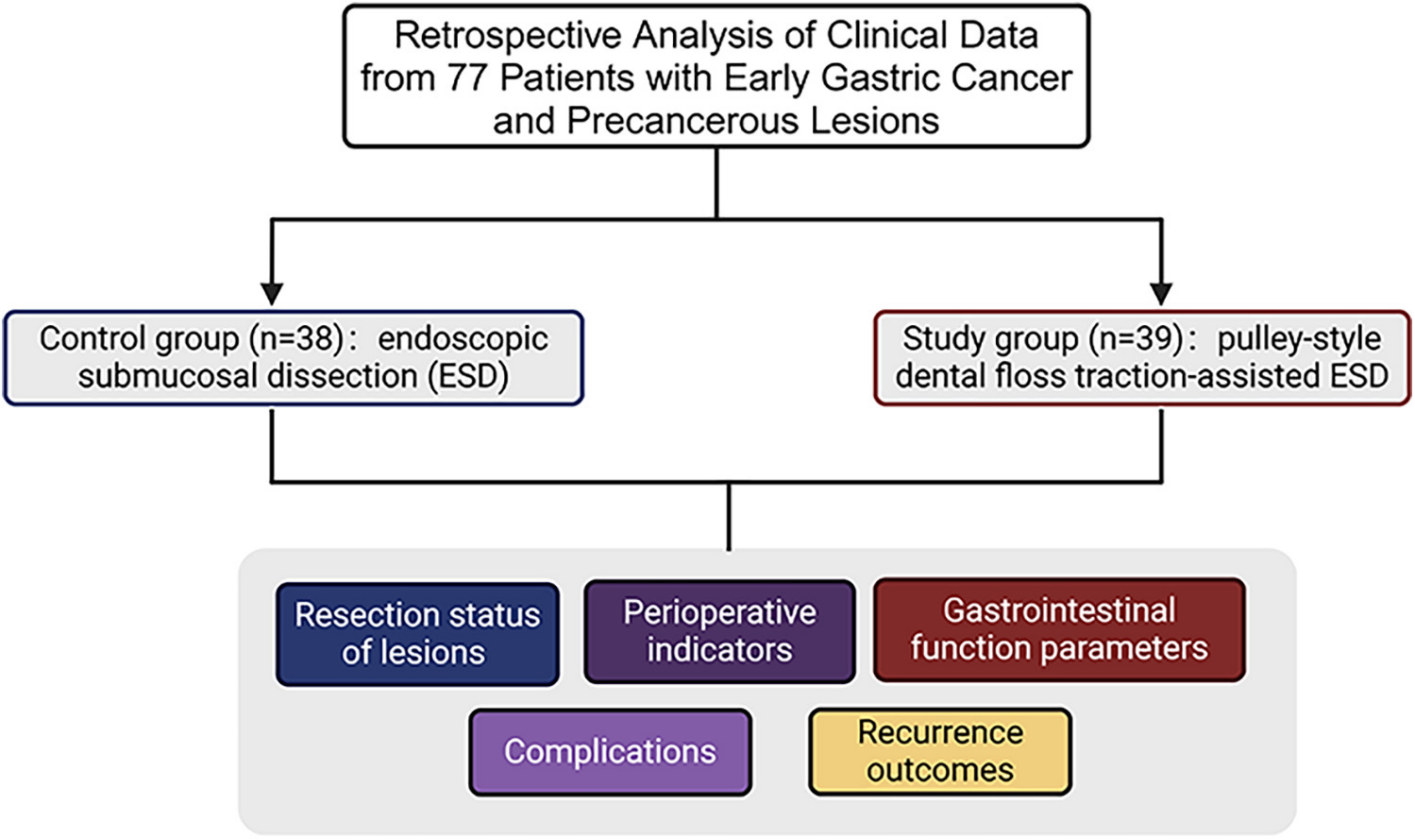
Flow diagram of the study design.

Clinical data of 77 patients with early gastric cancer and precancerous lesions treated at our hospital between February 2021 and June 2023 were retrospectively analyzed. Among these patients, 47 were male and 30 were female, aged between 38 and 74 years, with an average age of (55.69 ± 5.06) years. The lesion types included 50 EGC cases of early gastric cancer and 27 precancerous lesions. The body mass index (BMI) ranged from 16 to 30 kg/m^2^, with an average BMI of 21.52 ± 1.32 kg/m^2^. The lesion locations were as follows: gastric antrum in 35 patients, gastric corpus in 30 patients, and gastric fundus and cardia in 12 patients. According to the American Society of Anesthesiologists (ASA) classification, 33 and 44 patients were classified as grades I and II, respectively. The 77 patients with early gastric cancer and precancerous lesions were divided into control (*n* = 38) and a study groups (*n* = 39) based on different treatment protocols (Figure 1).

### Ethical statement

This study was conducted adherence to STROBE guidelines for observational studies and was approved by the Ethics Committee of the Shanghai Pudong New Area People's Hospital [(2022) Ethics Review No. (K87)]. All patients provided written informed consent before enrollment in the study.

### Eligible criteria

(1) Inclusion criteria: Compliance with the diagnostic standards for early gastric cancer and precancerous lesions (14), complete relevant clinical data, lesion diameter <30 mm, ASA grades I and II, surgical indications, age between 30 and 80 years, and expected survival time exceeding six months.
(2) Exclusion criteria: Concurrent with other types of malignancies, patients who received radiotherapy or chemotherapy prior to enrollment, distant metastasis of the tumor, pregnant and lactating women, and severe dysfunction of other organs.

## Methods

**Figure 2:**
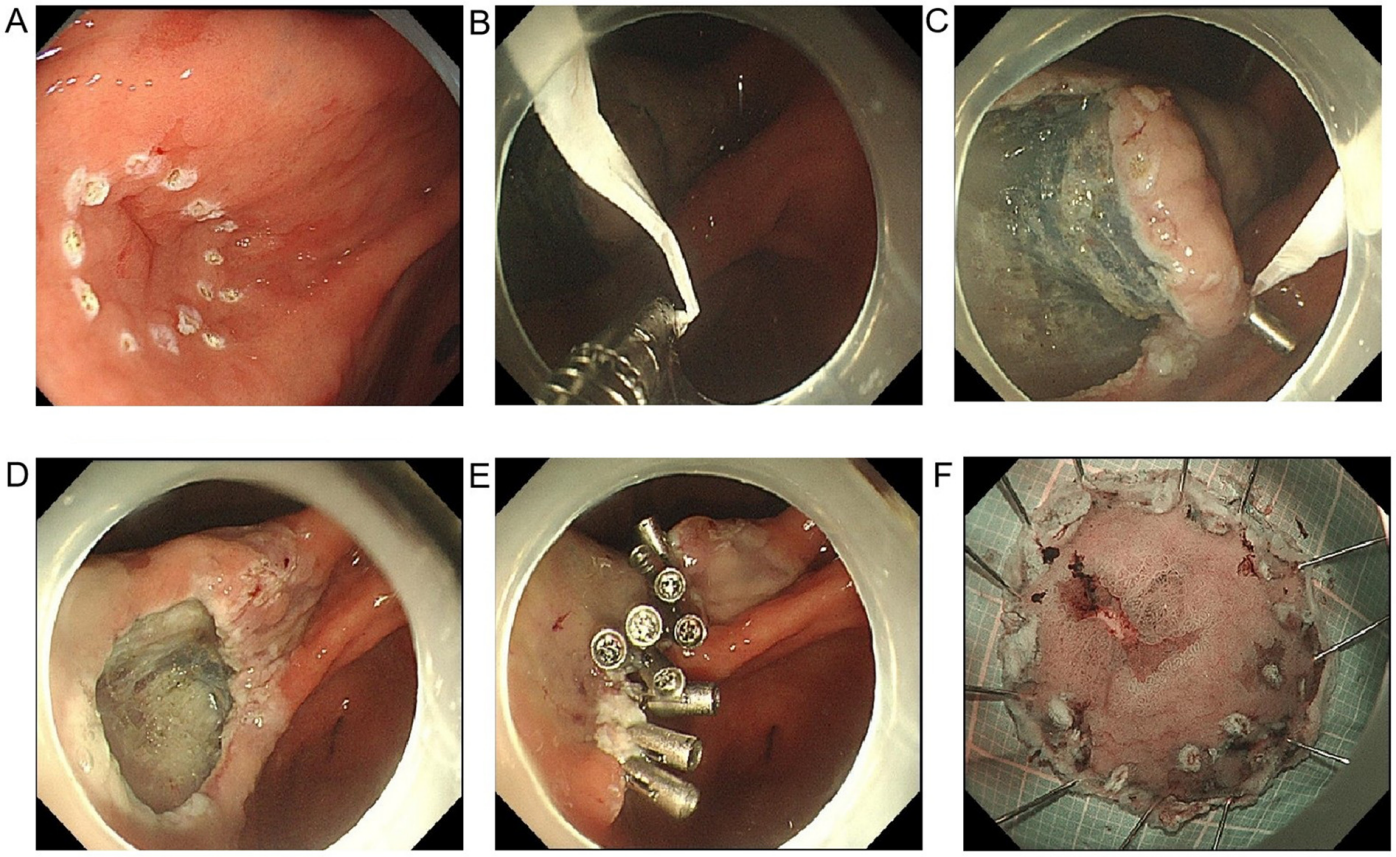
Image of floss traction-assisted ESD procedure. **(A)** Endoscopic identification of early gastric lesion: a localized elevation with clear boundaries and a granular or whitish surface is observed on the gastric mucosa, suggestive of early gastric cancer or high-grade intraepithelial neoplasia. The lesion extent is determined based on these features, which guide the marking process for subsequent resection; **(B)** construction of the pulley traction system: dental floss is secured to the arms of a hemostatic clip to form the basis for pulley-assisted traction, enabling precise manipulation and lesion exposure; **(C)** completion of the pulley mechanism and establishment of the traction pathway: the floss-equipped clip is attached to the lesion edge, with the floss exiting externally or through a sheath. By adjusting the traction angle, tissue tension is modulated, thereby forming a pulley-like mechanism under endoscopy, which significantly improves visualization and operational space; **(D)** exposure of the submucosal layer under traction: pulling the lesion outward with dental floss improves submucosal visualization, facilitating smooth execution of ESD. The wound shows well-defined margins and exposed vessels without active bleeding; **(E)** wound closure and hemostasis: multiple hemostatic clips are applied postoperatively to close the mucosal defect, reducing the risk of delayed bleeding or perforation. This approach is particularly effective for managing large mucosal defects safely; **(F)** en bloc resected specimen: the lesion is completely excised, displaying a well-defined margin and a red-white mottled surface. The specimen is pinned and stretched for pathological evaluation, reflecting the thoroughness and precision of the resection.

**Figure 3:**
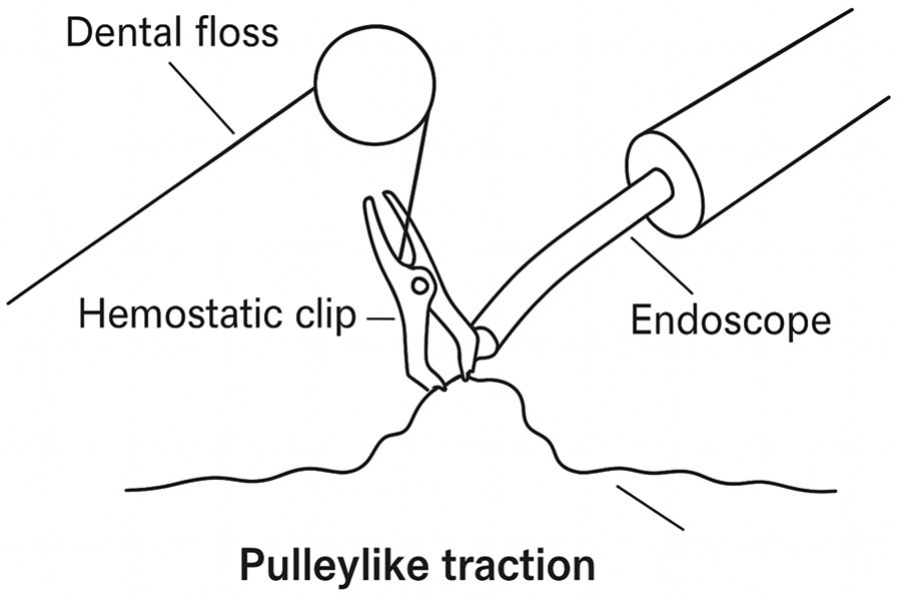
Construction of the pulley traction device. Under endoscopic guidance, the operator secures dental floss to a hemostatic clip and attaches it to the lesion. By altering the direction of traction, a pulley structure is formed, effectively increasing tissue tension to expose the submucosal layer and assist in performing ESD.

All patients underwent comprehensive examinations upon admission and preoperative fasting was performed according to regulations. The control group received ESD treatment in the left lateral position with general anesthesia administered via endotracheal intubation. Under endoscopic guidance, lesion location and extent were identified using 0.2% indigo carmine staining, followed by electrocoagulation marking. A submucosal injection solution with 1 ml of 2% adrenaline, 4 ml of 1% methylene blue, and 100 ml of 0.9% sodium chloride was prepared. After multi-point injections, the lesion tissue was separated from the muscle layer. The mucosa was incised along the electrocoagulation marks using repeated injections and separation procedures to ensure complete dissection of the lesion mucosa. A snare could be used to assist in the dissection. Electrocoagulation was performed for wound hemostasis, and titanium clips were used to seal areas of the gastric wall that were weakly or locally dissected deeper after resection. Pathological tissues were promptly sent for examination after surgery and the patients fasted for 24 h after surgery. The study group underwent pulley traction-assisted ESD with dental floss (Figure 2). Patients were placed in the left lateral decubitus position and general anesthesia was administered via endotracheal intubation. First, the lesion site was determined using endoscopy, followed by routine staining, marking, and lesion separation. After the mucosa of the lesion was incised, the endoscope was withdrawn and the biopsy channel was released. Next, hemostatic clip was threaded through the endoscopic channel to secure the floss. Thereafter, the endoscope was reinserted, and hemostatic clip was affixed to the dissected lesion area. Hemostatic clip was reinserted through the endoscopic channel to hook the dental floss, which was secured to the normal mucosal surface opposite and distal to the lesion. A gap was maintained between the hemostatic clip and mucosal layer to enable the floss to slide freely, forming a pulley traction system (Figure 3). External traction was applied with a certain force to pull up the lesion area, ensuring complete separation between the mucosal layer and the muscularis propria, thereby fully exposing the submucosal layer of the lesion. The mucosa of the lesion was peeled off in close proximity to the muscular layer. The remaining surgical steps were identical to those in the control group.

In this study, commercially available nylon dental floss (not a specialized medical material) was used as the traction tool. It possesses favorable flexibility and tensile strength, and is also easy to handle and cost-effective. When strict aseptic techniques are maintained intraoperatively, it can be safely utilized to assist with traction during ESD procedures.

### Observation indicators

The primary outcome measures included lesion resection status and postoperative recurrence. These indicators directly reflect the resection efficacy and disease control of traction-assisted ESD with dental floss and serve as core indicators for assessing therapeutic efficacy.

(1) Lesion resection. The curative resection rate refers to the proportion of cases in which the tumor is completely removed in a single procedure with intact tumor tissue, no vascular invasion, and both margins confirmed negative by endoscopic evaluation. The *en bloc* resection rate refers to the proportion of lesions completely excised, yielding a single, intact specimen. The residual basal lesion rate refers to the proportion of cases with residual lesion tissue present after surgery. The calculation of these indicators allowed the assessment of the efficacy of various treatment methods, particularly in terms of resection efficiency and tumor integrity.
(2) Recurrence. The patients were instructed to undergo regular follow-ups. All patients were followed up for 12 months and postoperative recurrence rates at 6 and 12 months were compared between the two groups. Recurrence was defined as the detection of new lesions on endoscopy.

Secondary outcome measures included perioperative indicators, gastrointestinal function indicators, incidence of complications, and quality of life. These indicators indirectly reflected the impact of treatment on the physical recovery, complication management, and quality of life of patients.

(1) Perioperative indicators. Intraoperative bleeding, operative time, anal exhaust time, first eating time, and length of hospital stay were compared between the two groups.
(2) Gastrointestinal function indicators. Fasting venous blood samples (3 ml) were collected from both groups preoperatively and three days postoperatively. The samples were centrifuged at 3,500 r/min for 10 min at a radius of 6 cm. The supernatant serum was transferred to 1.5 ml EP tubes and stored at −80°C for testing. Motilin (MTL) and gastrin (GAS) levels were measured using radioimmunoassay, whereas pepsinogen (PG I and PG II) levels were determined using ELISA. All reagent kits were provided by Shanghai Bioesn Biotechnology Co., Ltd.
(3) Complications. Complications including intestinal obstruction, pulmonary infection, delayed hemorrhage, and gastric perforation were compared between the two groups.
(4) Quality of Life. The quality of life in the two groups was assessed using the FACT-G scale preoperatively and three months postoperatively. This scale comprised four domains: emotional, functional, social/family, and physical wellbeing, each with a maximum score of 100 points. Quality of life was positively correlated with scores in each domain of the FACT-G.

### Statistical analysis

Statistical analysis software (SPSS 24.0) was used for the data processing. Continuous data (such as perioperative indicators, gastrointestinal function indicators, quality of life scores) were expressed as mean ± standard deviation $\left(\bar{\chi }\pm s\right)$ and analyzed using the *t*-test. Categorical data (lesion resection status, complications, and recurrence) were expressed as *n* (%) and analyzed using the *χ*^2^ test. Statistical significance was set at *P* < 0.05.

## Results

### Comparison of clinical data between the two groups

No significant differences were observed between the two groups in terms of lesion type, sex, age, lesion location, BMI, ASA classification, or other clinical data (*P* > 0.05) (Table 1).

**Table 1:** Comparison of clinical data between the two groups $n(\bar{\chi }\pm s)$.

Clinical data		Control group (*n* = 38)	Study group (*n* = 39)	*χ*^2^/*t*	*P*
Gender	Men	24	23	0.142	0.707
	Women	14	16		
Age (years)		55.2 ± 6.4	55.8 ± 5.9	0.428	0.670
Lesion types		23/15	27/12	0.641	0.424
BMI (kg/m^2^)		21.33 ± 2.06	21.87 ± 1.97	0.257	0.612
Lesion location	Gastric antrum	16	19	0.578	0.749
	Gastric corpus	15	15		
	Gastric fundus and cardia	7	5		
ASA classification	Grade I	15	18	0.351	0.554
	Grade II	23	21		

### Comparison of lesion resection status between the two groups

In the study group, the curative resection rate was 84.62% (33/39) and *en bloc* resection rate was 97.44% (38/39), which were higher than those [63.16% (24/38) and 78.95% (30/38), respectively] in the control group, whereas the residual rate of basal lesions in the study group was 2.56% (1/39), markedly lower than 23.68% (9/38) in the control group, exhibiting a significant difference (*P* < 0.05). This indicated that traction-assisted ESD with dental floss can improve the resection rate of early gastric cancer and precancerous lesions.

### Comparison of perioperative indicators between the two groups

**Figure 4:**
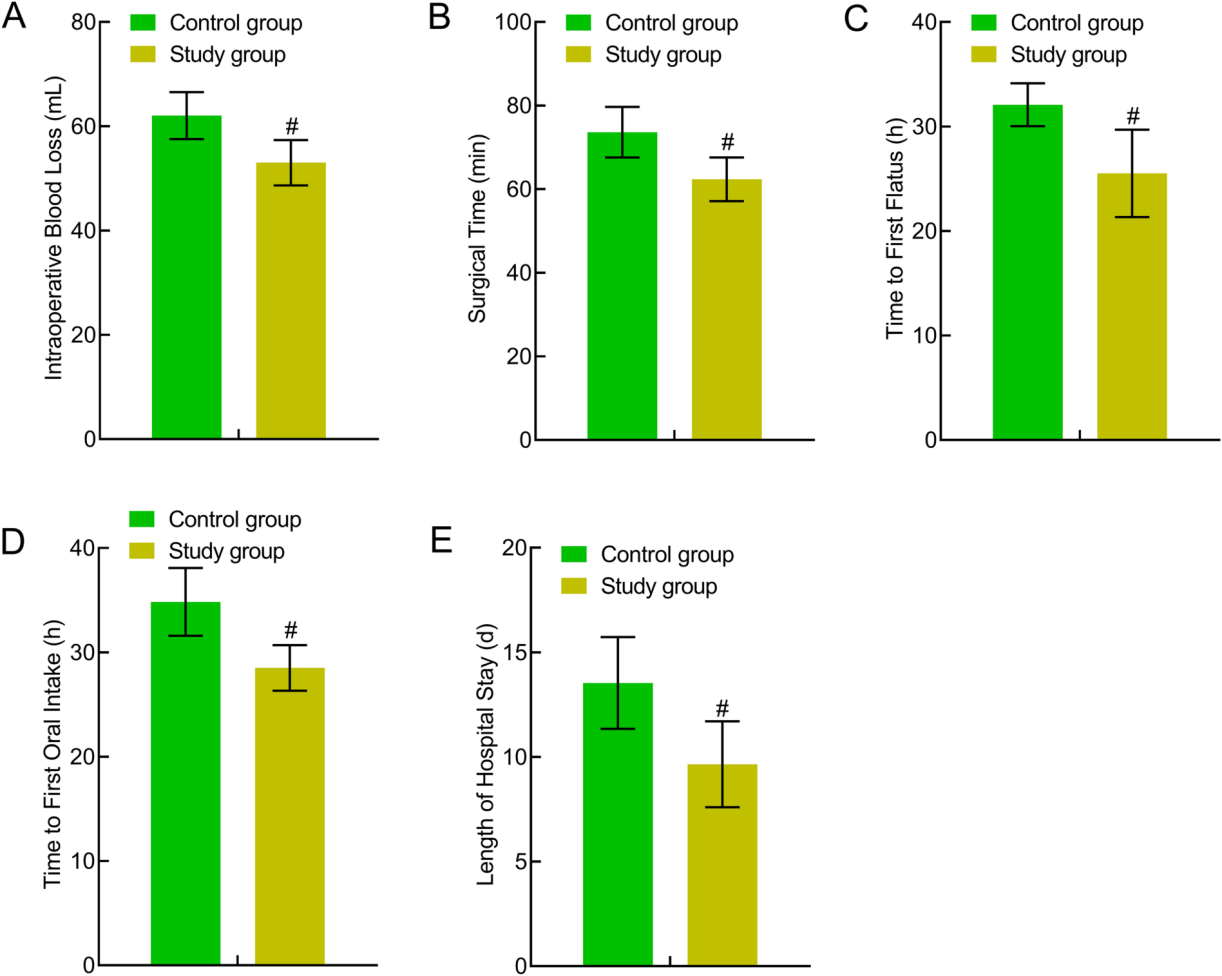
Comparison of perioperative indicators between the two groups. **(A)** Intraoperative bleeding; **(B)** operative time; **(C)** anal exhaust time; **(D)** first eating time; **(E)** Length of hospital stay. Compared to the control group, ^#^*P* < 0.05.

The study group experienced less intraoperative bleeding and had a significantly shorter operative time, anal exhaust time, first eating time, and length of hospital stay (*P* < 0.05). This indicated that traction-assisted ESD using dental floss can accelerate perioperative recovery in patients with early gastric cancer and precancerous lesions (Figure 4).

### Comparison of gastrointestinal function indicators between the two groups

**Figure 5:**
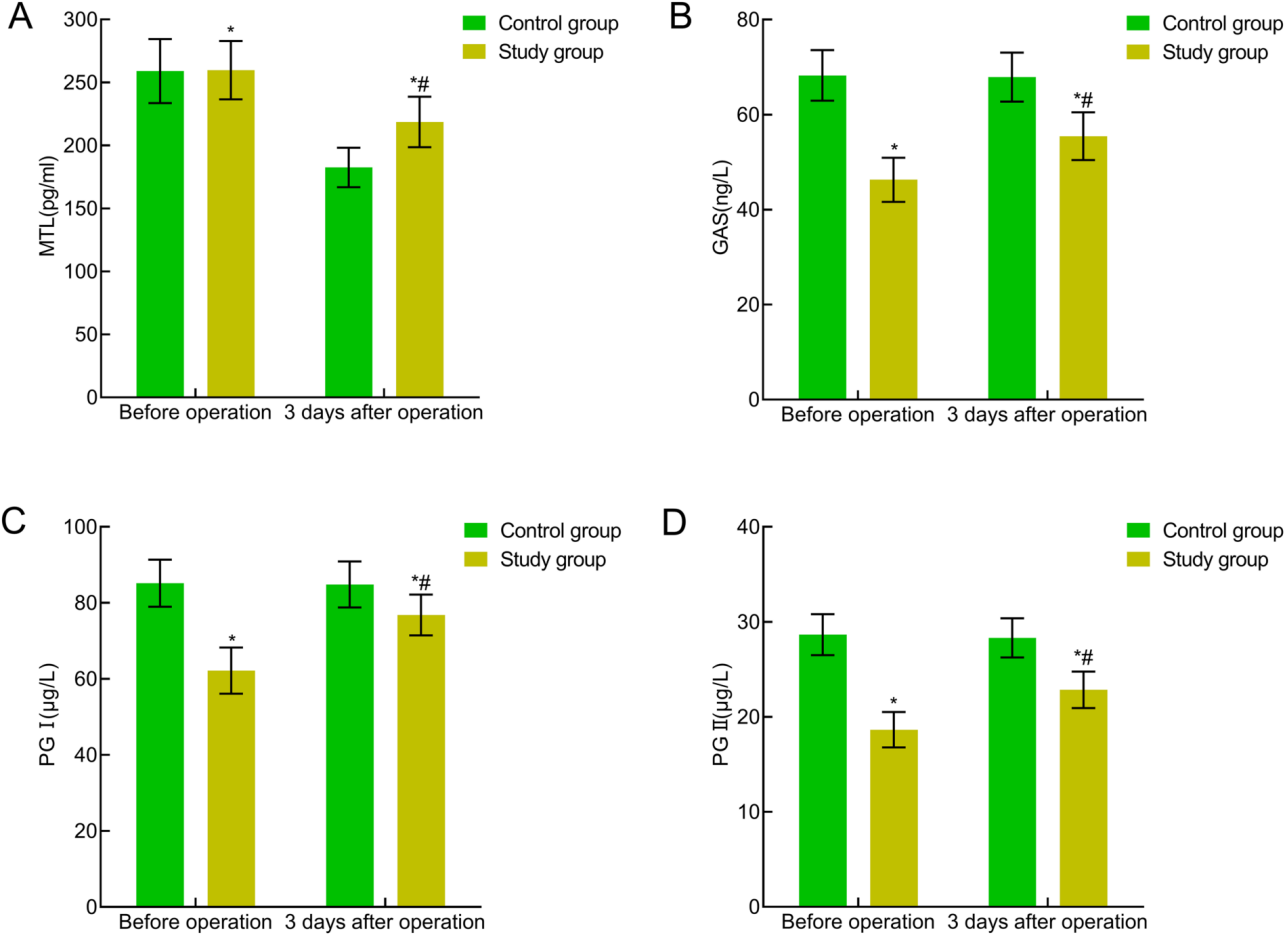
Comparison of gastrointestinal function indicators between the two groups. **(A)** MTL; **(B)** GAS; **(C)** PG I; **(D)** PG II. Compared with the preoperative period in this group, *P < 0.05; compared with the control group, ^#^*P* < 0.05.

No significant differences were present preoperatively between the two groups in terms of gastrointestinal function indicators (MTL, GAS, PG I, and PG II) (*P* > 0.05). Three days postoperatively, the MTL, GAS, PG I, and PG II levels were significantly reduced in both groups, with higher levels in the study group indicating a significant difference (*P* < 0.05). All four indicators showed statistical significance both before and after Bonferroni correction (all adjusted *P* < 0.05). The adjusted *P*-values remained significant, indicating the statistical robustness of the treatment group's advantage in gastrointestinal function recovery at postoperative day 3. This indicated that traction-assisted ESD with dental flossing can improve gastrointestinal function in patients with early gastric cancer and precancerous lesions (Figure 5).

### Comparison of complications between the two groups

In the control group, the incidence rates of intestinal obstruction, pulmonary infection, delayed hemorrhage, and gastric perforation were 5.26%, 5.26%, 10.53%, and 7.89%, respectively. In the study group, the corresponding incidence rates were 2.56%, 2.56%, 2.56%, and 0.00%, respectively. The study group demonstrated a significantly lower complication rate of 7.69% (3/39) compared to 26.32% (10/38) in the control group (*P* < 0.05).

### Comparison of quality of life between the two groups

**Figure 6:**
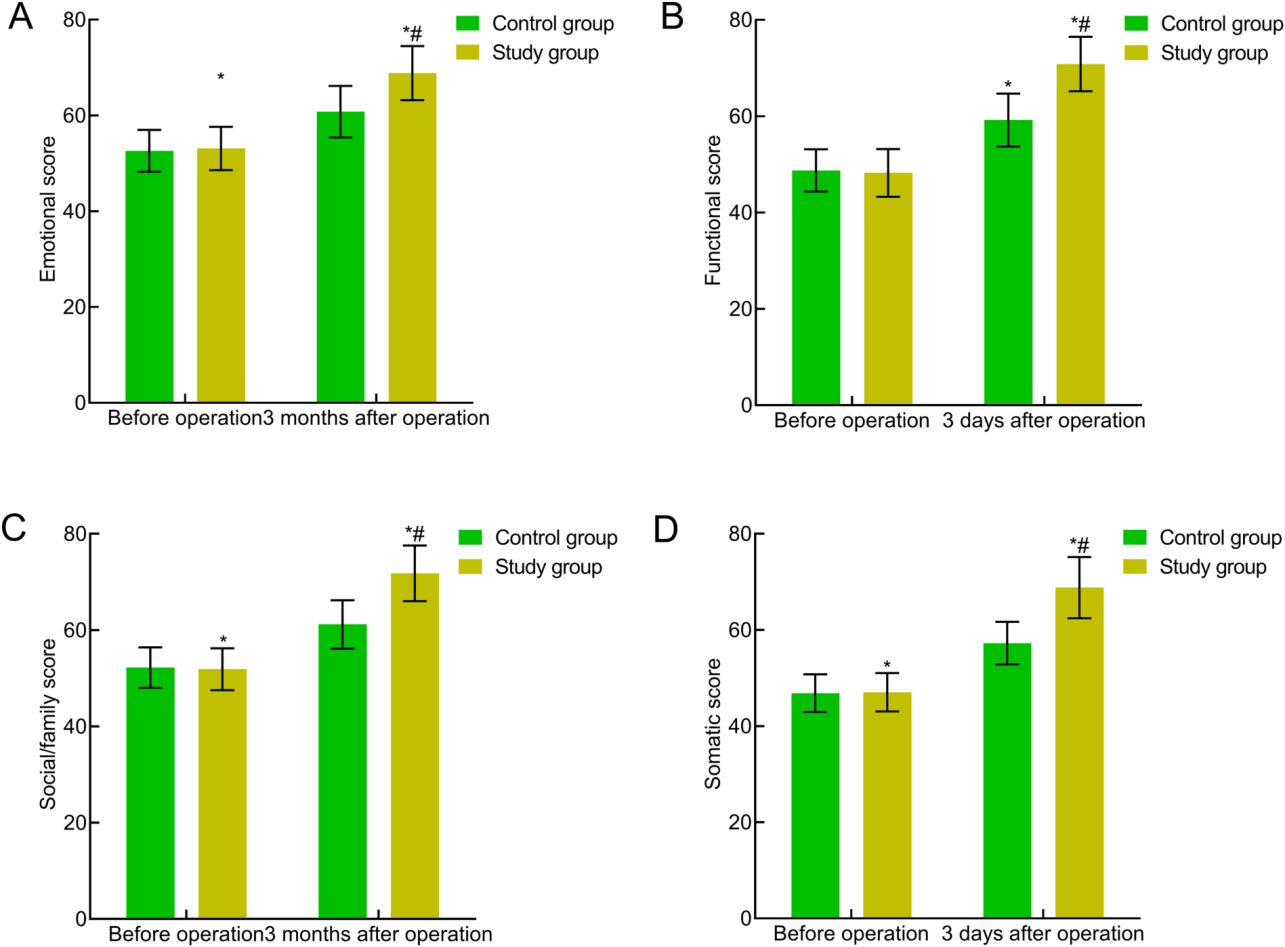
Comparison of quality of life between the two groups. **(A)** Emotional well-being; **(B)** functional well-being; **(C)** social/family well-being; **(D)** physical well-being. Compared with the preoperative period in this group, *P < 0.05; compared with the control group, ^#^*P* < 0.05.

The preoperative quality of life scores in all aspects of the FACT-G were comparable between the two groups (*P* > 0.05). Three months postoperatively, the quality of life scores in all aspects of the FACT-G significantly increased in both groups, with the study group showing significantly higher scores (*P* < 0.05). This indicated that traction-assisted ESD with dental flossing can significantly improve the quality of life of patients with early gastric cancer and precancerous lesions (Figure 6).

### Comparison of recurrence between the two groups

Six months postoperatively, the recurrence rate was 7.89% in the control group and 2.56% in the study group, with no statistically significant difference (*χ*^2^ = 0.292, *P* = 0.589). However, 12 months postoperatively, the recurrence rate in the study group (5.13%) was significantly lower than that in the control group (23.68%) (*χ*^2^ = 5.412, *P* = 0.020).

## Discussion

The results of this study showed that the lesion resection in the study group was superior to that in the control group, whereas the residual rate of basal lesions and the incidence of complications were lower than those in the control group. All the perioperative indicators were better in the study group and the quality of life scores in all aspects of the FACT-G of the two groups were elevated three months postoperatively, with the study group showing higher scores (*P* < 0.05). This indicated that pulley traction-assisted ESD with dental flossing is effective in promoting the recovery of patients with early gastric cancer and precancerous lesions, reducing complications, and enhancing their quality of life. The reasons can be attributed to the following aspects: Traction-assisted ESD with dental floss offers several advantages: (1) The materials used for traction-assisted with dental floss are lightweight, compact, and do not obstruct the endoscopic view, facilitating the dissection process. In addition, these materials are simple and readily available (15–17). (2) A combination of hemostatic clip and dental floss is used to secure the floss on the healthy mucosal surface opposite the lesion and ensure that it forms a pulley traction system. This method, using external traction forces to expose the lesion, may be particularly suitable for cases requiring finer control of traction and exposure. This allows complete visualization of the lesion mucosal field of view, facilitating complete excision of the lesion while minimizing the risk of perforation during dissection (18). (3) When removing a specimen, traction with a floss can effectively reduce tissue damage. However, owing to the limited tension of the dental floss, excessive pulling during the procedure may cause a floss break. Therefore, practitioners must possess a high level of proficiency in accurately controlling pulling force (19). To minimize traction-related complications, practitioners are advised to carefully control the traction angle and tension during the procedure. When necessary, the use of a tension gauge or team coordination to adjust the tension can help prevent dental floss break or tissue injury.

Clinical observations indicate that stimuli, such as anesthesia and surgical trauma can induce acute stress responses, activating the sympathetic-adrenal medullary system, leading to the release of substantial amounts of catecholamines, activating neutrophils and macrophages, and releasing various inflammatory cytokines. These processes trigger inflammatory responses, causing tissue hypoxia and ischemia, metabolic disorders, gastrointestinal dysfunction, and the release of gastrointestinal hormones, such as MTL, GAS, PG I, and PG II (20, 21). Monitoring these indicators (MTL, GAS, PG I, and PG II) is vital for assessing the recovery of gastrointestinal function. MTL is directly linked to gastrointestinal motility and its reduced secretion significantly impairs motility. GAS can enhance gastrointestinal function by reducing the pressure on the lower esophageal sphincter. PG I and PG II serve as crucial biomarkers of gastric mucosal health, reflecting gastric acid secretion and the number of gastric wall cells, respectively. In this study, these indicators served as objective evidence of the recovery of gastrointestinal function. This study showed that three days postoperatively, gastrointestinal function indicators (MTL, GAS, PG I, and PG II) were significantly reduced in both groups, with higher levels in the experimental group (*P* < 0.05). Pulley traction-assisted ESD with dental floss not only mitigates postoperative gastrointestinal dysfunction but also effectively accelerates the recovery of gastrointestinal hormones, facilitating a swifter restoration of gastrointestinal function. This may be due to fewer gastrointestinal trauma caused by traction-assisted ESD with dental floss, which effectively minimizes stress responses, prevents prolonged sympathetic nervous system excitation, alleviates inflammatory reactions, and mitigates the suppression of gastrointestinal function (22, 23). The improvement in these physiological indicators further confirmed the positive effect of pulley traction-assisted ESD with dental floss on the recovery of gastrointestinal function.

Additionally, this study revealed through follow-up that the recurrence rate in the study group 12 months postoperatively (5.13%) was significantly lower than that in the control group (23.68%) (*P* < 0.05). This suggests that pulley traction-assisted ESD with dental floss can achieve a favorable prognosis for early gastric cancer and precancerous lesions, probably due to complete exposure of the submucosal layer and a clearer surgical field, which facilitates complete lesion resection (24, 25). The novelty of this method lies in its emphasis on the free-pulley traction design of the floss. A combination of hemostatic clip and floss was used to secure the floss on a healthy mucosal surface opposite the lesion, ensuring that it formed a pulley traction system. This allowed external traction to expose the lesion, making the method potentially more suitable for cases requiring finer control of the traction force and lesion exposure. Therefore, although this method shared similarities with the previous method, it differed in the specific procedural steps and instruments utilized, with potentially greater precision in the distribution of traction and exposure of lesions. Through precise endoscopic traction, exposure of the lesion site was significantly enhanced, surgical trauma was minimized, and both the resection rate and postoperative recovery were effectively improved.

The pulley traction-assisted ESD with dental floss employed in this study offers advantages such as ease of operation, low cost, and readily available materials, making it particularly suitable for routine clinical settings with limited resources. In contrast, although the clip-with-line technique is widely used in gastrointestinal endoscopic surgery, it has limitations in wider luminal areas such as the gastric body or antrum. The restricted traction angles in these regions may affect mucosal tension control and resection efficiency, and frequent repositioning of the clip increases procedural complexity (26). Magnetic anchors can offer more flexible traction directions; however, they rely on specialized magnetic components and external control platforms, requiring advanced equipment and operator expertise, which limits their widespread clinical adoption (27). Robotic-assisted traction techniques (such as the EndoSAMURAI and MASTER systems) offer higher precision and dynamic control during procedures. However, their widespread use is limited by high costs, complex system structure, and steep technical requirements, making them primarily suitable for exploratory applications in advanced medical centers (28). Dental floss traction achieves stable lesion traction without the need for additional consumables or equipment, thereby improving surgical field exposure, enhancing procedural efficiency, and significantly reducing economic burden. Although it offers less flexibility in traction angle compared to magnetic or robotic systems, its simplicity, practicality, and good reproducibility make it more valuable for widespread application in routine ESD procedures.

Among various traction-assisted ESD techniques, the dental floss traction method offers a significant cost-effectiveness advantage. Compared to commercially available clip-with-line systems, magnetic anchors, and robotic-assisted equipment that require additional purchases, dental floss is inexpensive and widely accessible, enabling effective traction without increasing medical costs. This makes it especially suitable for primary care hospitals or resource-limited settings. Moreover, dental floss traction is relatively simple to perform and does not rely on expensive or complex equipment, facilitating its adoption in routine endoscopy units. Although it has some limitations in adjusting traction angles, its benefits in cost control, instrument availability, and ease of implementation make it a practical technique suitable for widespread clinical application.

This study has several limitations. First, as a single-center retrospective study with a relatively small sample size, the generalizability of the findings may be limited. Second, the retrospective design inherently carries risks of bias, such as selection bias and operator skill variability, which may affect intraoperative efficiency, complication rates, and recurrence assessments. Although we adhered to objective criteria in data collection and analysis and controlled for major confounding factors through multivariable analysis, outcome assessors were not completely blinded to patient grouping information documented in medical records. This may have introduced observational bias, particularly in outcomes involving subjective judgment such as recurrence and complication assessments. Furthermore, although a 12-month follow-up was conducted and demonstrated preliminary short-term efficacy, longer-term data (e.g., 3–5 years) are lacking, limiting a comprehensive evaluation of the long-term effects of pulley traction-assisted ESD with dental floss on recurrence control and survival outcomes. Therefore, future studies should involve larger sample sizes, implement rigorous blinding, and adopt multicenter, prospective, randomized controlled trial designs with extended follow-up periods to more comprehensively assess the long-term efficacy and safety of this technique. Moreover, the long-term prognosis following pulley traction-assisted ESD with dental floss has not yet been systematically evaluated, and relevant data remain insufficient. To enhance the reliability and generalizability of the findings, future multicenter, large-sample prospective studies are warranted. Such studies should incorporate stratified analyses (e.g., based on operator experience) and rigorous blinding methods to improve study quality, reduce potential bias, and further validate the mid- to long-term efficacy and safety of the technique demonstrated in this study.

## Conclusions

The pulley traction-assisted ESD with dental floss demonstrated remarkable efficacy in treating early gastric cancer and precancerous lesions, significantly enhancing gastrointestinal function recovery, reducing postoperative complications and recurrence rates, enhancing quality of life, and ensuring a favorable prognosis.

## Data Availability

The raw data supporting the conclusions of this article will be made available by the authors, without undue reservation.
